# Temporal trends in hyperuricaemia in the Irish health system from 2006-2014: A cohort study

**DOI:** 10.1371/journal.pone.0198197

**Published:** 2018-05-31

**Authors:** Arun Kumar A. U., Leonard D. Browne, Xia Li, Fahd Adeeb, Fernando Perez-Ruiz, Alexander D. Fraser, Austin G. Stack

**Affiliations:** 1 Department of Nephrology, University Hospital Limerick, Limerick, Ireland; 2 Graduate Entry Medical School, University of Limerick, Limerick, Ireland; 3 Department of Mathematics and Statistics, La Trobe University, Melbourne, Australia; 4 Department of Rheumatology, University Hospital Limerick, Limerick, Ireland; 5 Rheumatology Division, Hospital Universitario Cruces, University of the Basque Country, Bilbao, Vizcaya, Spain; 6 Health Research Institute (HRI), University of Limerick, Limerick, Ireland; Shanghai Diabetes Institute, CHINA

## Abstract

**Background:**

Elevated serum uric acid (sUA) concentrations are common in the general population and are associated with chronic metabolic conditions and adverse clinical outcomes. We evaluated secular trends in the burden of hyperuricaemia from 2006–2014 within the Irish health system.

**Methods:**

Data from the *National Kidney Disease Surveillance Programme* was used to determine the prevalence of elevated sUA in adults, age > 18 years, within the Irish health system. Hyperuricaemia was defined as sUA > 416.4 μmol/L in men and > 339.06 μmol/L in women, and prevalence was calculated as the proportion of patients per year with mean sUA levels above sex-specific thresholds. Temporal trends in prevalence were compared from 2006 to 2014 while general estimating equations (GEE) explored variation across calendar years expressed as odds ratios (OR) and 95% Confidence intervals (CI).

**Results:**

From 2006 to 2014, prevalence of hyperuricaemia increased from 19.7% to 25.0% in men and from 20.5% to 24.1% in women, P<0.001. The corresponding sUA concentrations increased significantly from 314.6 (93.9) in 2006 to 325.6 (96.2) in 2014, P<0.001. Age-specific prevalence increased in all groups from 2006 to 2014, and the magnitude of increase was similar for each age category. Adjusting for baseline demographic characteristics and illness indicators, the likelihood of hyperuricemia was greatest for patients in 2014; OR 1.45 (1.26–1.65) for men and OR 1.47 (1.29–1.67) in women vs 2006 (referent). Factors associated with hyperuricaemia included: worsening kidney function, elevated white cell count, raised serum phosphate and calcium levels, elevated total protein and higher haemoglobin concentrations, all P<0.001.

**Conclusions:**

The burden of hyperuricaemia is substantial in the Irish health system and has increased in frequency over the past decade. Advancing age, poorer kidney function, measures of nutrition and inflammation, and regional variation all contribute to increasing prevalence, but these do not fully explain emerging trends.

## Introduction

Serum uric acid (sUA) has emerged as an important biomarker of cardiovascular health and a large body of evidence now incriminates elevated concentrations in the development of several chronic metabolic conditions, cardiovascular disease, and associated mortality [1–6]. Prospective epidemiological studies have demonstrated that rising sUA concentrations are independently associated with the development of chronic kidney disease, new-onset hypertension, and type 2 diabetes [3–6]. Moreover, evidence has accumulated that elevated sUA concentrations above conventional thresholds predict future myocardial infarction, stroke and cardiovascular death and all-cause mortality [7–9]. Collectively, these studies suggest at the very least that sUA is an important metabolic and cardiovascular biomarker that merits measurement and surveillance.

Given the potential contribution of sUA to chronic disease and mortality, periodic surveillance of sUA concentrations at a population level and within health systems is desirable to evaluate burden and temporal trends [10–12]. A study from the US by Zhu *et al* found significantly higher burden of hyperuricaemia in men and in women in 2007–2008 compared to 1988–1994 that was partially attributed to increasing levels of obesity and hypertension [10]. A further study from Italy by Trifiro *et al* covering the period 2005–2009 reported a similar pattern [11]. In contrast, Chuang et al found that mean sUA levels decreased between 1993–1996 and 2005–2008 in Taiwan with a corresponding fall in burden of hyperuricaemia among men and women [12]. The lack of concordance across studies would suggest that the prevalence of hyperuricaemia varies substantially worldwide and that country-to-country differences exist possibly reflecting differences in underlying genetic, dietary and lifestyle factors. There are limited studies that have described temporal trends in hyperuricaemia among patients who are captured within the health system [11]. Moreover, even fewer have investigated underlying reasons for these trends and whether longitudinal patterns in sUA concentrations might be related to changing demographic and clinical phenotypes.

In view of these knowledge deficits, we explored temporal trends in hyperuricaemia from 2006 to 2014 among patients within the Irish health system. Our primary objective was to examine patterns in temporal trends and ascertain whether any observed variation might be explained by changing demographic profiles, clinical measures of health status or geography.

## Methods

### Dataset

**Fig 1:**
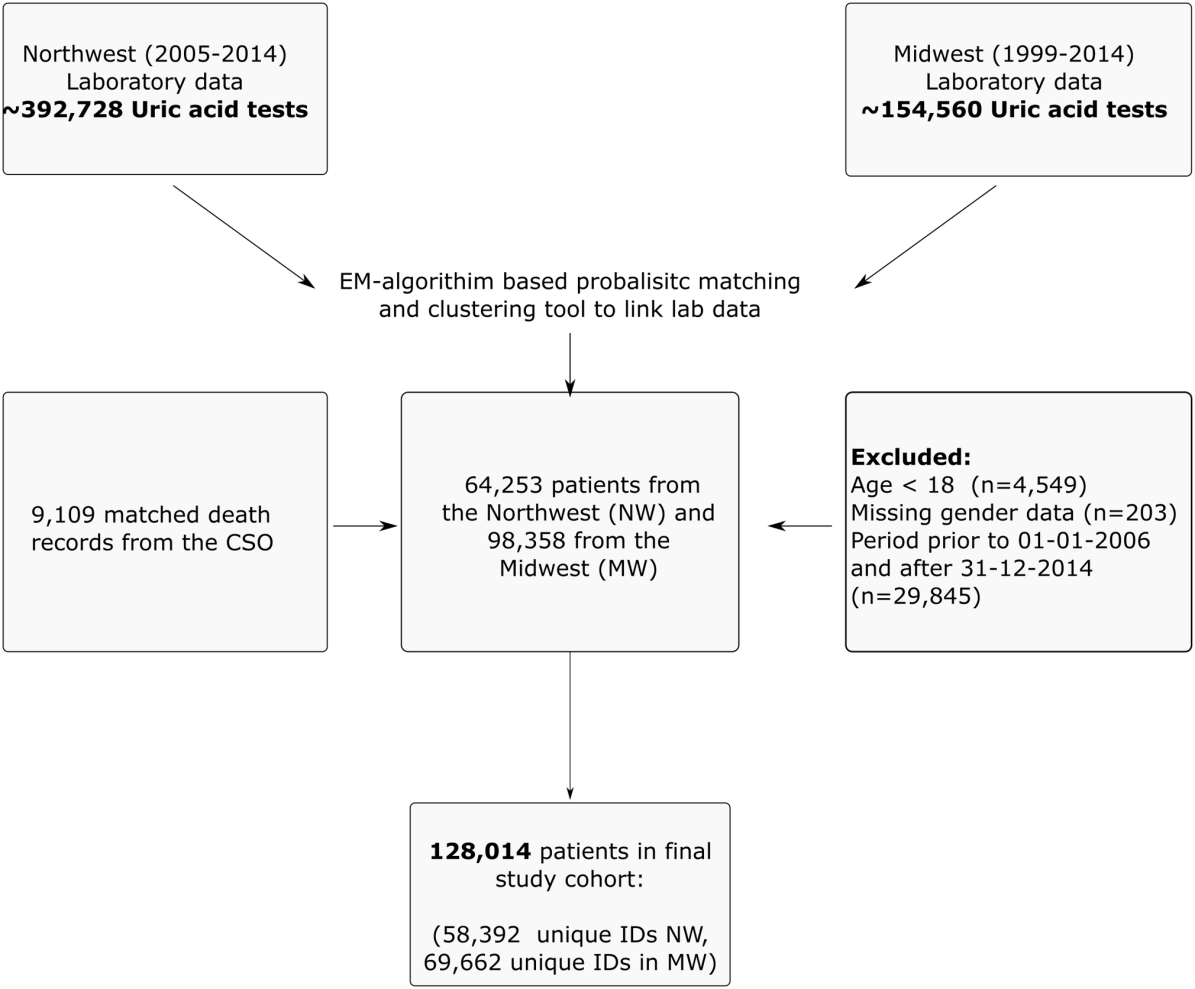
Strobe diagram for the retrospective cohort study from 2006–2014. The final dataset captured information on demographic characteristics, county of residence, primary location of patient supervision, measured laboratory parameters, dialysis indicator variables and death.

We utilised data from the *National Kidney Disease Surveillance System* which serves to monitor trends and outcomes of kidney disease in the Irish health system [13]. The system integrates and links health system data from multiple sources across large provincial regions in the Irish health system through a secure network. The principal data sources include: regional laboratory information systems which capture both inpatient and outpatient laboratory tests within a designated region, dialysis registers which capture incident dialysis; and mortality data files from the national Central Statistics Office (CSO). We identified all patients with measured sUA concentrations values from two major health regions; Northwest region (from 2005–2011) and Midwest region (from 1999–2013), and linked laboratory data records over time using an EM-algorithm based probabilistic matching strategy [14]. Excluding missing data on age, sex, and unmatched mortality records, we identified 128,014 patients with linked demographic, laboratory and outcome data (Fig 1).

### Laboratory data

An enzymatic colorimetric method using uricase enzyme on an auto analyzer was used to measure sUA concentrations during which uric acid is converted to allantoin and H_2_O_2_. Serum creatinine was measured using the modified kinetic Jaffe method and creatinine values were calibrated to be traceable to an isotope dilution mass spectrometry (IDMS) reference measurement procedure to ensure standardization. Serum creatinine values were used to estimate glomerular filtration rate (eGFR) in ml/min per 1.73 m^2^ using the Chronic Kidney Disease Epidemiology Collaboration (CKD-EPI) [15]. Chronic Kidney Disease (CKD) was defined according to the Kidney Disease Dialysis Quality Outcome Initiative (KDOQI) guidelines based on eGFR measurements. Data was captured on an extensive list of laboratory measures including indicators of inflammation (c-reactive protein, ESR, white cell count and subtypes), nutrition (serum albumin and total protein), bone biomarkers (serum calcium and phosphate), and haemoglobin. The location of medical supervision was defined as the location where the first uric acid test was ordered by the supervising health professional and categorized as; inpatient location (IP), outpatient department (OP), general practice (GP) and emergency room (ER). County of residence was available for each patient and allowed us to classify patients by geography. The principal counties served by the Northwest region of Ireland included Donegal, Sligo, Leitrim and Roscommon while those served by the Midwest region included Limerick, Clare and Tipperary.

### Definition of hyperuricaemia

Hyperuricaemia was defined as sUA > 416.4 μmol/L in men and > 339.06 μmol/L in women respectively. These values were chosen based on the laboratory definition as utilised in NHANES 3 [10] and have been widely used in the literature. Our definition was based on a single sUA for each patient within a specific year. For patients with more than one sUA value per year, the mean sUA was determined. The values of sUA are reported in micromoles per litre and can be converted to milligrams per decilitre by dividing by 59.48.

### Statistical analysis

Descriptive statistics were calculated for continuous variables (reported as mean values and standard deviations or median and IQR where appropriate) and categorical variables (reported as numbers and percentages). Differences between categories were evaluated using the Kruskal Wallis test, Fisher’s Exact test or the χ2 test, as appropriate.

The prevalence of hyperuricemia was calculated as the proportion of patients within a calendar year with a mean sUA; > 416.4 μmol/L in men and > 339.06 μmol/L in women respectively among all patients with a measured sUA concentration. Prevalence was calculated according to age and sex distributions, location of medical supervision, country of residence, and among clinical subgroups according to eGFR, and glycosylated haemoglobin (≥ 6.5% vs less). Temporal trends in prevalence of hyperuricaemia were determined and compared across years using chi-square proportional trend test.

General Estimating Equations (GEE) with a logit link function for binary outcomes and an exchangeable correlation structure were fitted to explore the associations of demographic, clinical and geographic factors with the prevalence of hyperuricaemia. The sandwich estimator was used to adjust the standard errors of the estimated coefficients to account for the longitudinal aspect of the design. A marginal (‘population-average’) regression model was selected to analyse the average prevalence associated with each characteristic while accounting for within-subject correlation over time. In a series of sequentially adjusted models we assessed whether the association of calendar year with hyperuricaemia could be explained by demographic, clinical or geographic factors. Calendar year was modelled primarily as a categorical variable with 2006 as referent. A final multivariable model was constructed to identify the relative contributions of demographic, clinical and health system factors with the presence of hyperuricemia, thereby clarifying the temporal relationship. The associations of explanatory factors with hyperuricaemia were represented by adjusted odds-ratios (AOR) and 95% CI. Wald statistics from the corresponding generalized estimating equations were used to test for the significance of these associations. The interactions between age and sex were tested in multivariable models for risk of hyperuricaemia.

The correlation information criterion (CIC) as proposed by Hin and Wang was used to determine correlation structures for repeated measurements [16]. The discriminative capacity of the models was determined by the Area Under the Curve (AUC) value of the Receiver Operating Characteristic (ROC) curves. All analyses were performed with the geepack, pROC packages using R statistical software.

### Sensitivity analyses

A set of sensitivity analyses explored the robustness of our observations. First, we examined to what extent our estimates were influenced by lowering our threshold definition of hyperuricaemia to sUA > 356.8 μmol/L (6.0 mg/dl) for both men and women based on evidence derived from expert panels ascertaining risk thresholds [17,18]. Second, we considered the impact of sUA > 416.4 μmol/L (7.0 mg/dl) for men and women, a threshold value above the supersaturation point of sUA. Finally, we substituted the mean for the median sUA and repeated the entire analysis to determine whether this would materially alter our estimates.

### Ethics approval

Ethical approval and a waiver of informed consent for the study were granted by the Ethics Committee at University Hospital Limerick. All patient records were fully anonymised prior to data analysis which was conducted at the Data Coordinating Centre (DCC).

## Results

### Baseline characteristics of the study cohort

There were 128,014 individuals, age 18 years or older, who had valid sUA measurements from 2006–2014. Of these 25,497 (19.9%) were defined as hyperuricaemic at first entry into the health system. The mean age was 52.4 (17.7) years, and 51. 5% were women (Table 1). Patients with hyperuricaemia were significantly older; predominantly men, had higher serum creatinine values, inflammatory markers, and had significantly lower estimated GFR values compared to those without hyperuricaemia (all P < 0.001).

**Table 1:** Patient characteristics at baseline by presence or absence of hyperuricaemia^1^.

Variable	N	No Hyperuricaemia	Hyperuricaemia	P-value
Count (%)	128,014	102,517 (80.1)	25,497 (19.9)	
Age [Mean(SD)]	128,014	51.0 (17.3)	58.1 (18.1)	<0.001
**Sex**				
% Female	65,862	52.14	48.69	
% Male	62,152	47.86	51.31	<0.001
**Age Group (n, %)**				
18–39 years	34,687	29.13	18.9	
40–59 years	46,337	37.42	31.26	
60–80 years	39,440	28.94	38.31	
>80 years	7,550	4.50	11.52	<0.001
**County of Residence (n, %)**				
Donegal	26,177	21.58	15.9	
Sligo	18,140	14.76	11.8	
Leitrim	7,971	6.57	4.86	
Roscommon	796	0.63	0.57	
Limerick	37,425	27.89	34.63	
Clare	12,818	9.69	11.31	
Tipperary	6,255	4.58	6.12	
Other Counties	18,432	14.3	14.81	<0.001
**Location of Medical Supervision**^2^				
Emergency Room (ER)	4,769	3.74	4.55	
General Practice (GP)	86,951	71.81	68.73	
Inpatient (IP)	15,816	12.57	14.43	
Outpatient (OP)	14,608	11.88	12.29	<0.001
**Laboratory Variables**^3^				
**Measures of Kidney Function**				
Creatinine (μmol/L)	108,444	71.7 (21.0)	84.0 (30.3)	<0.001
eGFR (ml/min/1.73m^2^)	108,444	95.0 (26.8)	78.9 (38.3)	<0.001
Urea (mmol/L)	110,664	5.6 (2.2)	6.7 (3.1)	<0.001
**eGFR Category**^4^				
eGFR ≥90	61,736	62.16	35.39	
eGFR 60–89	36,798	32.79	38.62	
eGFR 30–59	8,585	4.65	21.35	
eGFR 15–29	1,056	0.28	3.83	
eGFR < 15	269	0.11	0.81	<0.001
**Measures of Inflammation**				
C-Reactive Protein (mg/L)	15,083	3.0 (4.0)	5.0 (9.9)	<0.001
ESR (mm/hr)	41,854	10.2 (14.0)	14.8 (21.6)	<0.001
White blood count (x10^9^/L)	100,381	6.7 (4.2)	7.4 (4.0)	<0.001
Lymphocyte count(x10^9^/L)	98,492	1.7 (1.0)	1.7 (1.0)	0.001
Neutrophil count (x10^9^/L)	99,951	3.9 (3.2)	4.4 (3.2)	<0.001
**Measures of Nutrition**				
Serum Albumin (g/L)	95,613	38.2 (5.1)	37.4 (5.5)	<0.001
Total protein (mmol/L)	89,594	68.3 (5.9)	69.0 (6.6)	<0.001
**Measures of Bone Metabolism**				
Serum Calcium (mmol/L)	66,251	2.3 (0.1)	2.3 (0.1)	<0.001
Serum Phosphate (mmol/L)	56,378	1.1 (0.2)	1.2 (0.3)	<0.001
**Metabolic Biomarkers**				
Serum Uric Acid (umol/L) [Mean(SD)]	128,014	280.7 (65.0)	448.1 (80.5)	<0.001
Haemoglobin (g/dl)	100,384	13.3 (1.8)	13.3 (2.0)	<0.001
Ferritin (ng/ml)	38,123	120.2 (264.5)	180.0 (259.1)	<0.001
Glucose (mmol/L)	23,385	5.0 (1.0)	5.2 (1.2)	<0.001
Serum Potassium (mmol/L)	89,446	4.3 (0.5)	4.4 (0.5)	<0.001
HbA1c (%)	10,530	5.8 (1.4)	6.0 (1.1)	<0.001
**Glycaemic Control (n, %)**				
HbA1c <6.5%	7,546	71.7	71.5	
HbA1c ≥6.5%	2,984	28.3	28.5	0.879
**Lipids**				
Total Cholesterol (mmol/L)	46,250	5.1 (1.1)	5.2 (1.2)	<0.001
Triglyceride (mmol/L)	50,536	1.2 (0.9)	1.6 (1.2)	<0.001
High Density Lipoprotein (mmol/L)	9,430	1.3 (0.4)	1.2 (0.4)	<0.001
Low Density Lipoprotein (mmol/L)	31,359	3.2 (0.9)	3.3 (1.0)	<0.001

^1^ Hyperuricaemia was defined as sUA > 416.4 μmol/L in men and > 339.06 μmol/L in women respectively based on the laboratory definition from NHANES 3 [10].

^2^ Location of medical supervision refers to the location of patient when the laboratory test was conducted.

^3^ Values are reported as median with interquartile range

^4^ eGFR: Estimated glomerular filtration rate (ml/min per 1.73 m^2^) was based on the Chronic Kidney Disease Collaborative (CKD-EPI) equation [15]

### Prevalence of hyperuricaemia between 2006–2014

**Fig 2:**
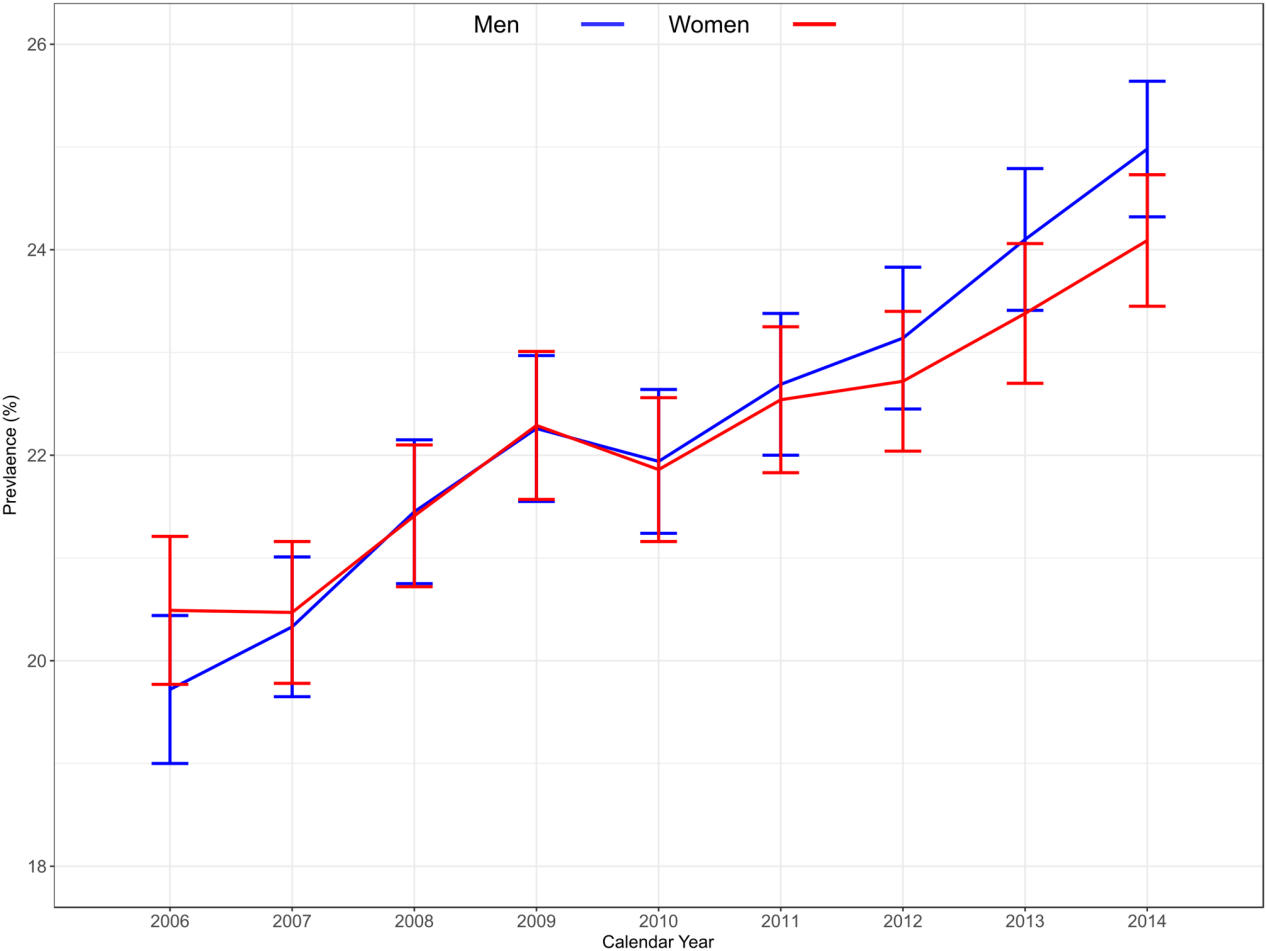
Temporal trends in the prevalence of hyperuricaemia by sex in the health system. Error bars represent 95% CI’s calculated form the direct method.

**Fig 3:**
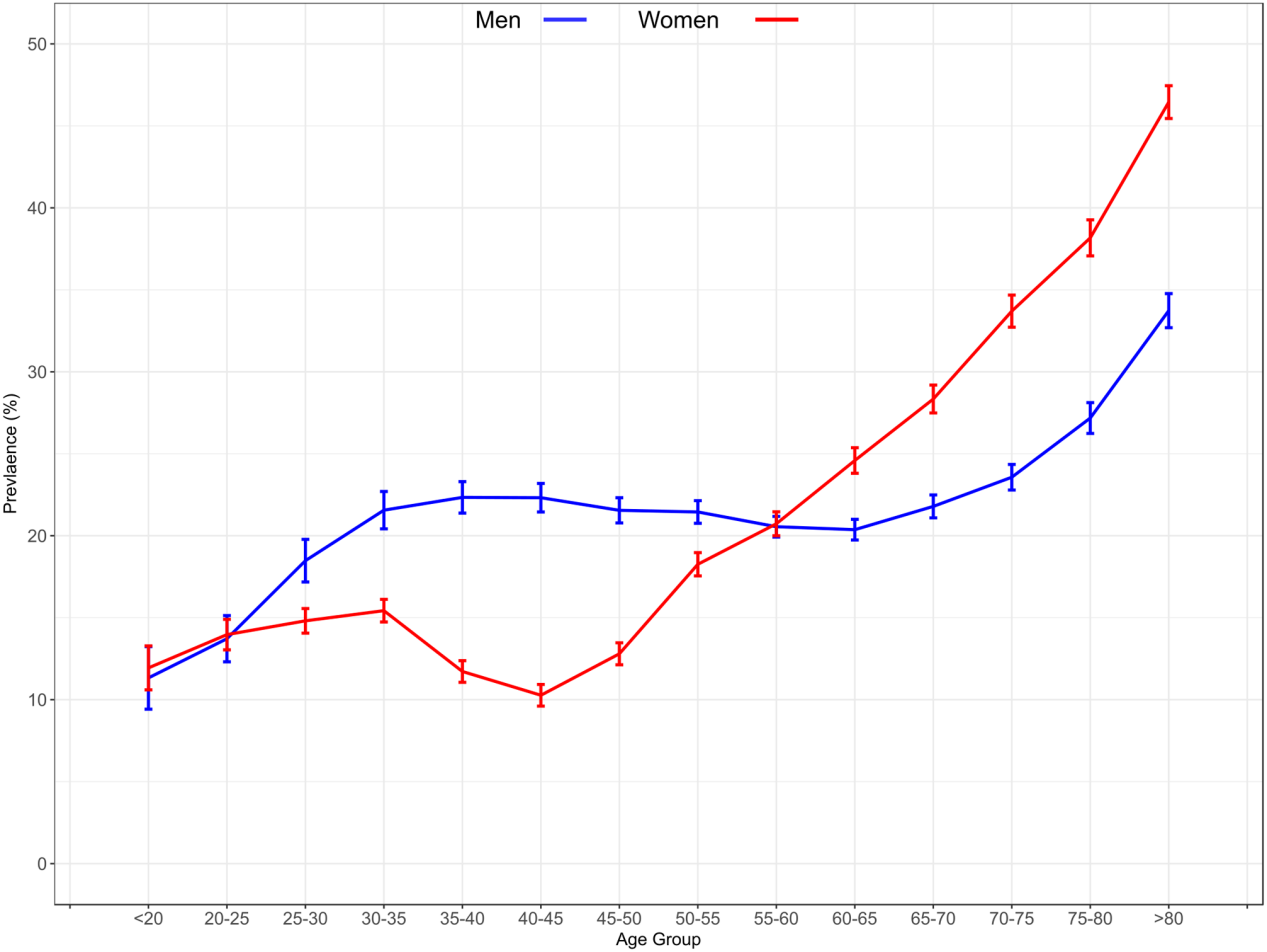
Temporal trends in the prevalence of hyperuricaemia by age and sex in the Irish health system. Error bars represent 95% CI’s calculated form the direct method.

Table 2 shows the temporal trends in prevalence of hyperuricaemia from 2006 to 2014. Overall prevalence of hyperuricaemia increased from 20.1% in 2006 to 24.5% in 2014 and this pattern was present for both men and women (Fig 2, P<0.001 for each). Age-specific prevalence was observed to increase in all groups from 2006 to 2014, and the magnitude of increase was similar for each age category. Although the overall prevalence of hyperuricaemia was higher in men than women, this was not consistent across age categories as shown in Fig 3. From young adulthood to middle-age, the prevalence of hyperuricaemia was higher in men than women; however, after the age of 60 years, the prevalence of hyperuricaemia rose sharply in women and far exceeded that of men.

**Table 2:** Temporal trends in prevalence of hyperuricaemia in the Irish health system.

Variable	2006	2007	2008	2009	2010	2011	2012	2013	2014	P-value
Events of hyperuricaemia Uric Acid [Mean(SD)^1^]	4776 314.6 (93.9)	5425 316.0 (93.2)	5760 319.3 (93.5)	5796 322.0 (93.9)	5852 321.6 (92.4)	6246 323.1 (92.9)	6603 322.9 (93.8)	7038 324.8 (94.9)	8289 325.6 (96.2)	<0.001
**Age group**										
18–39 years	13.7 (12.8, 14.7)	13.5 (12.6, 14.4)	15.2 (14.2, 16.1)	16.7 (15.7, 17.7)	15.4 (14.4, 16.4)	15.9 (14.9, 16.8)	16.2 (15.3, 17.2)	17.2 (16.3, 18.2)	18.6 (17.6, 19.5)	<0.001
40–59 years	16.5 (15.7, 17.3)	17.6 (16.8, 18.3)	18.0 (17.3, 18.8)	19.1 (18.3, 19.9)	18.7 (17.9, 19.4)	18.5 (17.7, 19.2)	19.3 (18.5, 20.1)	20.4 (19.6, 21.2)	20.2 (19.5, 21)	<0.001
60–80 years	24.1 (23.2, 25)	23.9 (23.1, 24.8)	25.0 (24.2, 25.9)	25.5 (24.6, 26.4)	25.1 (24.2, 25.9)	26.6 (25.8, 27.5)	26.3 (25.5, 27.2)	27.0 (26.2, 27.8)	27.7 (26.9, 28.4)	<0.001
>80 years	39.2 (36.8, 41.6)	38.2 (36, 40.4)	39.8 (37.6, 42)	39.9 (37.6, 42.3)	40.8 (38.5, 43.1)	42.3 (40.1, 44.6)	41.7 (39.5, 43.8)	40.2 (38.1, 42.3)	43.0 (41.1, 45)	0.001
**County of Residence**										
Donegal	15.7 (14.6, 16.9)	16.6 (15.5, 17.6)	18.5 (17.4, 19.6)	19.4 (18.3, 20.5)	19.2 (18.1, 20.3)	20 (18.9, 21.1)	20.5 (19.5, 21.6)	20.6 (19.5, 21.6)	20.8 (19.8, 21.8)	<0.001
Sligo	21.0 (19.7, 22.2)	18.9 (17.7, 20)	18.2 (17.1, 19.3)	19.8 (18.6, 21)	18.1 (17, 19.3)	22.6 (21.3, 24)	21 (19.7, 22.4)	21 (19.6, 22.5)	23.9 (22.4, 25.4)	<0.001
Leitrim	16.2 (14.5, 17.9)	15.4 (13.8, 16.9)	15.4 (13.9, 16.9)	17.2 (15.6, 18.9)	15.3 (13.6, 17)	19.2 (17.5, 21)	18.3 (16.8, 19.9)	16.3 (14.6, 17.9)	19.2 (17.4, 21)	0.001
Roscommon	16.7 (11, 22.3)	14.0 (9.2, 18.8)	14.0 (8.9, 19.1)	14.3 (9.1, 19.5)	17.9 (12.2, 23.6)	17.0 (11.6, 22.5)	22.3 (16, 28.6)	22.2 (15.5, 28.8)	25.2 (17.8, 32.6)	0.001
Limerick	22.6 (21.6, 23.5)	24.1 (23.2, 25)	26.2 (25.3, 27.2)	26.0 (25.1, 27)	25.5 (24.6, 26.4)	24.8 (23.9, 25.7)	25.8 (24.9, 26.6)	26.8 (25.9, 27.7)	27.9 (27.1, 28.8)	<0.001
Clare	21.2 (19.5, 22.8)	23.0 (21.3, 24.6)	23.5 (21.8, 25.2)	24.3 (22.6, 26)	23.6 (21.9, 25.2)	23.3 (21.8, 24.7)	24.1 (22.5, 25.7)	24.9 (23.4, 26.4)	25.3 (23.8, 26.8)	<0.001
Tipperary	22.5 (18.6, 26.5)	24.9 (21.7, 28.1)	25.3 (21.8, 28.7)	28.3 (24.9, 31.6)	23.6 (20.9, 26.3)	23.3 (20.7, 25.9)	24.2 (21.8, 26.7)	26.3 (24.3, 28.4)	25.5 (24, 26.9)	0.383
Other Counties	19.3 (17.9, 20.7)	18.8 (17.5, 20.1)	20.4 (19.1, 21.7)	21.1 (19.7, 22.5)	23.2 (21.8, 24.6)	22.2 (20.9, 23.6)	22.8 (21.4, 24.1)	24.5 (23.2, 25.8)	23.5 (22.4, 24.5)	<0.001
**Hospital region**										
Midwest	22.1 (21.4, 22.9)	23.4 (22.7, 24.1)	25.1 (24.4, 25.8)	25.4 (24.7, 26.1)	25.0 (24.3, 25.7)	24.1 (23.5, 24.8)	25.0 (24.3, 25.6)	26.0 (25.4, 26.7)	26.1 (25.5, 26.7)	<0.001
Northwest	17.7 (17, 18.5)	17.0 (16.3, 17.6)	17.6 (16.9, 18.2)	18.8 (18.1, 19.4)	18.0 (17.3, 18.7)	20.6 (19.9, 21.3)	20.2 (19.5, 20.9)	20.0 (19.3, 20.8)	21.5 (20.7, 22.2)	<0.001
**Location of Medical Supervision**^2^										
Emergency Room (ER)	24.0 (20.4, 27.5)	27.1 (23.7, 30.6)	27.8 (24.5, 31.2)	25.7 (22.6, 28.8)	22.5 (19.7, 25.4)	22.3 (19.6, 25)	26.6 (23.8, 29.5)	24.2 (21.1, 27.2)	23.6 (20.8, 26.5)	0.187
General Practice (GP)	19.3 (18.7, 19.9)	19.6 (19, 20.2)	20.7 (20.1, 21.3)	21.8 (21.2, 22.4)	21.8 (21.2, 22.5)	22.4 (21.8, 23)	22.1 (21.5, 22.7)	23.4 (22.8, 24)	23.8 (23.3, 24.3)	<0.001
Inpatient (IP)	22.8 (21.3, 24.2)	22.6 (21.3, 23.9)	23.0 (21.5, 24.4)	23.2 (21.7, 24.7)	22.3 (20.9, 23.8)	23.0 (21.6, 24.5)	23.2 (21.8, 24.7)	22.5 (21.1, 23.9)	24.5 (23, 26)	0.237
Outpatient (OP)	22.0 (20.3, 23.7)	24.2 (22.6, 25.8)	25.2 (23.7, 26.6)	24.7 (23.3, 26.1)	22.8 (21.5, 24.1)	24.0 (22.7, 25.3)	26.7 (25.3, 28)	27.3 (25.9, 28.6)	29.3 (27.9, 30.6)	<0.001
**Glycosylated Haemoglobin**^3^										
HbA1c <6.5%	27.8 (25.4,30.2)	23.5 (21.5,25.5)	26.9 (24.9,28.9)	29.3 (27.1,31.6)	27.5 (25.1,29.9)	26.0 (24.2,27.7)	24.0 (22.4,25.5)	25.7 (24.2,27.1)	26.0 (24.9,27.1)	0.218
HbA1c ≥6.5%	24.0 (20.6,27.4)	25.7 (22.6,28.7)	26.2 (23.5,28.9)	27.1 (24.3,29.8)	25.5 (22.6,28.3)	28.5 (26.1,31)	31.0 (28.5,33.4)	34.1 (31.6,36.7)	31.5 (29.4,33.6)	<0.001
**eGFR Category** ^4^										
eGFR 90	11.5 (10.8, 12.1)	11.1 (10.5, 11.7)	12.9 (12.3, 13.5)	13.8 (13.2, 14.5)	13.5 (12.9, 14.1)	13.6 (13, 14.1)	13.8 (13.2, 14.4)	14.8 (14.2, 15.4)	15.3 (14.7, 15.9)	<0.001
eGFR 60–89	19.2 (18.3, 20.1)	20.3 (19.5, 21.2)	22.8 (21.9, 23.7)	22.7 (21.8, 23.6)	23.0 (22.1, 23.9)	24.8 (23.9, 25.7)	23.7 (22.9, 24.6)	25 (24.2, 25.9)	25.1 (24.3, 25.9)	<0.001
eGFR 30–59	48.3 (46.1, 50.6)	48.4 (46.3, 50.4)	54.8 (52.8, 56.9)	54.4 (52.4, 56.3)	54.3 (52.4, 56.3)	54.9 (53, 56.8)	55.9 (54.1, 57.7)	53.8 (52, 55.5)	54.8 (53.2, 56.4)	<0.001
eGFR 15–29	75.2 (69.8, 80.6)	66.8 (61.5, 72.1)	73.0 (67.9, 78.1)	69.2 (64.3, 74.2)	66.2 (61.4, 71.1)	70.0 (65.5, 74.4)	70.5 (66.2, 74.8)	68.9 (64.8, 73)	67.7 (63.7, 71.7)	0.209
eGFR <15	59.2 (48.2, 70.3)	57.3 (46.1, 68.5)	48.7 (37.3, 60)	45.8 (35.1, 56.5)	38.0 (27.3, 48.7)	43.2 (32.4, 54)	45.6 (33.8, 57.4)	47.3 (35.9, 58.7)	48.9 (38.8, 59.1)	0.085

^1^ Prevalence of hyperuricemia was calculated as the proportion of patients within a calendar year with a mean sUA; > 416.4 μmol/L in men and > 339.06 μmol/L in women with 95% Confidence Intervals

^2^ Location of medical supervision refers to the location of patient when the laboratory test was conducted.

^3^ Haemoglobin A1c reported according to the Diabetes Control and Complications Trial (DCCT). Values reported as International Federation of Clinical Chemistry (IFFC) were converted to DCCT using the equation: IFCC-HbA1c (mmol/mol) = [DCCT-HbA1c (%)−2.15] x 10.929.

^4^ eGFR: Estimation of glomerular filtration rate (ml/min per 1.73 m^2^) was based on the Chronic Kidney Disease Collaborative (CKD-EPI) Equation [15]

### Prevalence of hyperuricaemia between 2006–2014: Regional and county variation

The prevalence of hyperuricaemia varied significantly across regions within the health system. In 2006, the prevalence was highest in the Midwest region (Counties of Limerick, Clare and Tipperary) at 22.1% compared to 17.7% in the Northwest region (Counties of Donegal, Sligo, Leitrim and Roscommon). Increases in prevalence were observed in each region over time but the relative differences persisted. At the county level, significant increases in the prevalence of hyperuricaemia were observed over time except in county Tipperary.

### Prevalence of hyperuricaemia by level of kidney function and by glycosylated haemoglobin (HbA1c)

**Fig 4:**
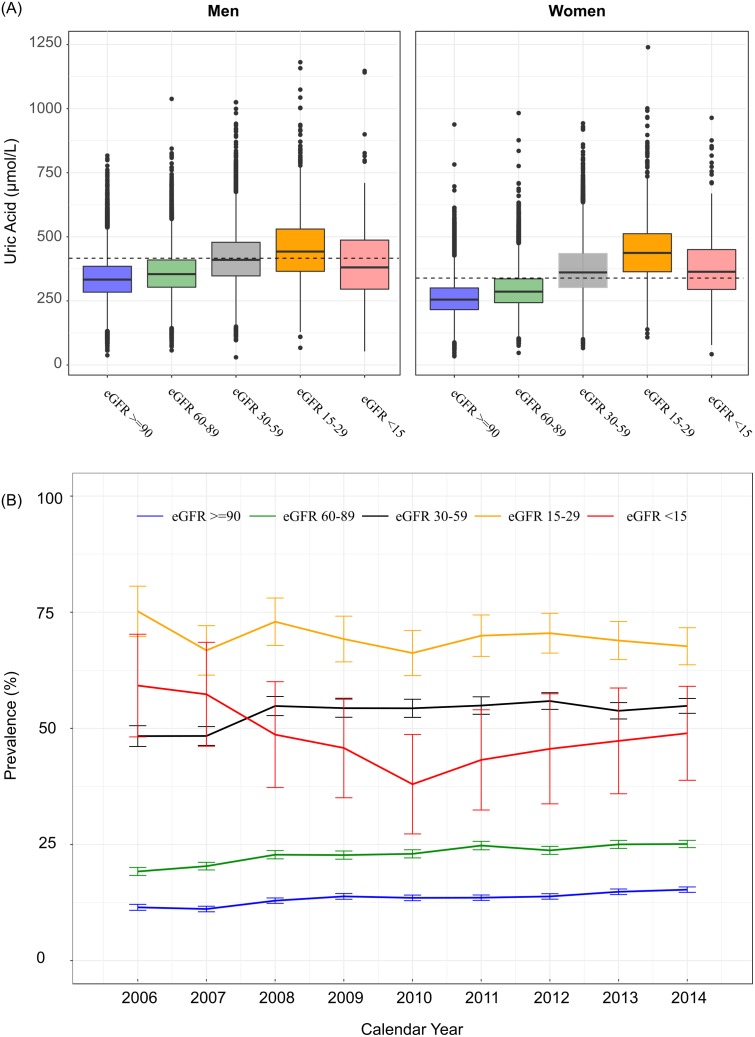
Temporal trends in the prevalence of hyperuricaemia by level of kidney function. (A) Illustrates the distribution of serum uric acid by eGFR category among men and women, the dashed line represents the respective threshold for hyperuricaemia. (B) Temporal trends in prevalence of hyperuricaemia in the health system from 2006–2014 by eGFR category.

The prevalence of hyperuricaemia increased significantly with worsening renal function, from 12.2% in patients with eGFR > 90ml/min to 63.9% in patients with eGFR < 15ml/min. The corresponding mean sUA levels increased from 297.2 (81.5) μmol/L in eGFR category 1 to 391.8 (146.7) μmol/L in eGFR category 5, although levels were highest for patients in eGFR category 4 at 450.3 (129.8) μmol/L. Among patients with mild–moderate kidney impairment (correlating with eGFR categories 1–3), significant trends of increasing prevalence were observed from 2006–2014 as shown in Fig 4. In contrast, among patients with severe kidney impairment (correlating with eGFR categories 4–5), the prevalence remained relatively stable over the same period. At baseline, the prevalence of hyperuricaemia was similar among patients with or without good glycaemic control (28.3% and 28.5% for HbA1c < 6.5% vs ≥ 6.5% respectively). However, from 2006 to 2014, the burden of hyperuricaemia among patients with HbA1c ≥ 6.5% increased by 7.5% from 24.0% (95% CI: 20.6%-27.4%) to 31.5% (95% CI: 29.4%–33.6%), P< 0.001.

### Patient-level factors associated with hyperuricaemia

**Fig 5:**
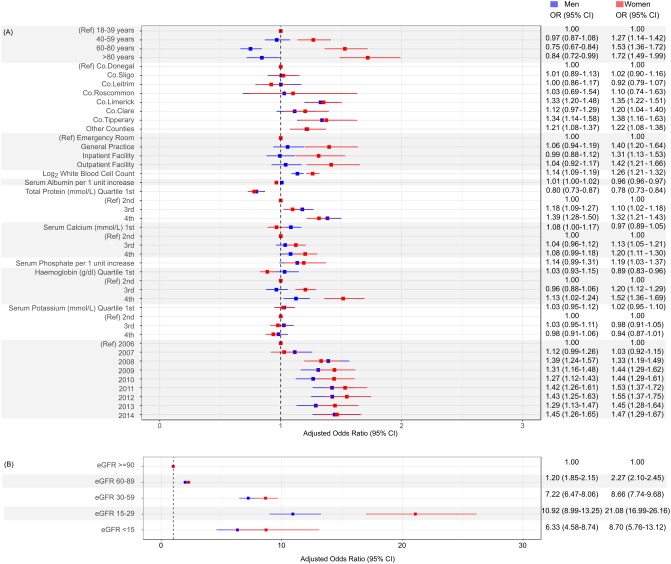
Forest plot of adjusted odds ratio of hyperuricaemia stratified by sex. Covariates for adjustment include: age group, eGFR CKD-stage in categories, county of residence, location of medical supervision, laboratory measures of illness (haemoglobin, serum albumin, white blood cell count, serum potassium, serum calcium and serum phosphate concentration) and calendar year with referent 2006. (A) Illustrates odds ratios for all covariates except CKD stage which is illustrated separately in (B).

Table 3 describes the relationship of demographic, clinical and geographic factors with hyperuricaemia within the health system. The likelihood of hyperuricaemia increased with advancing age category, multivariable OR, 1.27 (1.15–1.42), P< 0.01 for oldest vs youngest. A significant age-sex interaction (P<0.001) indicated that the likelihood of hyperuricaemia differed between men and women across age categories. Adjusting for calendar year only, the odds ratio of hyperuricaemia was significantly higher in older women than in older men. In the fully adjusted model, the likelihood of hyperuricaemia increased with age in women but not in men (Fig 5A).

**Table 3:** Factors associated with prevalent cases of hyperuricaemia from 2006–2014 in the Irish health system.

Variables	Unadjusted	Robust SE	P-value	Final Multivariable Model^1^	Robust SE	P-value
**Age Group**						
18–39 years	1.00			1.00		
40–59 years	1.21 (1.17, 1.26)	0.018	0.000	1.12 (1.03–1.2)	0.039	0.005
60–80 years	1.75 (1.69, 1.81)	0.018	0.000	1.09 (1.01–1.19)	0.042	0.034
>80 years	3.12 (2.96, 3.28)	0.026	0.000	1.27 (1.15–1.42)	0.054	<0.001
**Gender**						
Women	1.00			1.00		
Men	1.05 (1.02, 1.07)	0.013	0.000	0.77 (0.73–0.81)	0.027	<0.001
**County of Residence**						
Donegal	1.00			1.00		
Sligo	1.20 (1.02–1.42)	0.085	0.029	1.03 (0.94–1.12)	0.044	0.532
Leitrim	1.05 (0.98–1.11)	0.032	0.157	0.95 (0.85–1.06)	0.056	0.375
Roscommon	1.77 (1.66–1.88)	0.032	0.000	1.09 (0.83–1.44)	0.142	0.544
Limerick	1.76 (1.69–1.82)	0.020	0.000	1.34 (1.24–1.44)	0.038	<0.001
Clare	1.66 (1.58–1.75)	0.025	0.000	1.15 (1.04–1.28)	0.053	0.007
Tipperary	1.14 (1.08–1.19)	0.024	0.000	1.36 (1.21–1.53)	0.061	<0.001
All other counties	1.48 (1.41–1.54)	0.023	0.000	1.22 (1.13–1.33)	0.043	<0.001
**Location of Medical Supervision**						
Emergency Room (ER) (Reference)	1.00			1.00		
General Practice (GP)	0.93 (0.89–0.99)	0.027	0.012	1.17 (1.06–1.28)	0.048	0.001
Inpatient (IP)	1.02 (0.96–1.07)	0.028	0.586	1.12 (1.02–1.22)	0.048	0.024
Outpatient (OP)	1.00 (0.95–1.06)	0.029	0.995	1.18 (1.07–1.30)	0.048	0.001
**Laboratory Variables**						
**GFR Category (ml/min/1.73m**^**2**^**)**						
eGFR > = 90	1.00			1.00		
eGFR 60–89	1.89 (1.84–1.95)	0.014	<0.001	2.12 (2.01–2.24)	0.027	<0.001
eGFR 30–59	5.93 (5.70–6.17)	0.020	<0.001	8.03 (7.43–8.68)	0.039	<0.001
eGFR 15–29	12.20 (11.08–13.43)	0.049	<0.001	14.93 (12.98–17.17)	0.071	<0.001
eGFR <15	6.83 (5.65–8.25)	0.097	<0.001	7.58 (5.91–9.73)	0.127	<0.001
			<0.001			
**Inflammatory markers**			<0.001			
Log _2_(White blood count)	1.23 (1.2, 1.25)	0.010	<0.001	1.20 (1.16–1.24)	0.016	<0.001
Serum Albumin (per 1 g/L increase)	0.99 (0.98, 0.99)	0.001	<0.001	0.99 (0.98–0.99)	0.003	<0.001
**Nutritional/Metabolic Markers**						
Total protein (mmol/L) 1^st^ (<64.5)	0.96 (0.93–1.00)	0.017	0.031	0.81 (0.76–0.85)	0.028	<0.001
Total protein (mmol/L) 2^nd^ (64.5–68.7) *(Reference)*	1.00			1.00		
Total protein (mmol/L)3^rd^ (68.0–71.3)	1.10 (1.07, 1.13)	0.015	<0.001	1.14 (1.08–1.20)	0.026	<0.001
Total protein (mmol/L) 4^th^ (>71.3)	1.30 (1.26, 1.35)	0.017	<0.001	1.36 (1.29–1.44)	0.029	<0.001
Serum Calcium (mmol/L) 1^st^ (<2.25)	1.04 (1.00–1.08)	0.019	0.039	1.02 (0.96–1.07)	0.028	0.578
Serum Calcium (mmol/L) 2^nd^ (2.25–2.31) *(Reference)*	1.00			1.00		
Serum Calcium (mmol/L) 3^rd^ (2.31–2.38)	1.11 (1.07–1.15)	0.017	<0.001	1.09 (1.04–1.15)	0.026	0.001
Serum Calcium (mmol/L) 4^th^ (>2.38)	1.29 (1.25–1.34)	0.019	<0.001	1.17 (1.1–1.23)	0.029	<0.001
			<0.001			
Serum Phosphate (per 1 mmol/L increase)	1.42 (1.33–1.53)	0.037	<0.001	1.14 (1.04–1.26)	0.051	0.008
Haemoglobin (g/dl) 1st <12.1	1.15 (1.1, 1.19)	0.018	<0.001	0.96 (0.91–1.02)	0.031	0.205
Haemoglobin (g/dl) 2^nd^ 12.1–13.3 *(Reference)*	1.00			1.00		
Haemoglobin (g/dl) 3^rd^ 13.3–14.44	1.05 (1.02, 1.09)	0.017	0.002	1.13 (1.07–1.19)	0.029	<0.001
Haemoglobin (g/dl) 4^th^ >14.44	1.2 (1.16, 1.24)	0.018	<0.001	1.42 (1.33–1.52)	0.034	<0.001
Serum Potassium (mmol/L) 1^st^ (<4.07)	0.94 (0.90–0.97)	0.018	<0.001	1.03 (0.97–1.09)	0.028	0.337
Serum Potassium (mmol/L) 2^nd^ (4.07–4.3) *(Reference)*	1.00			1.00		
Serum Potassium (mmol/L) 3^rd^ (4.3–4.67)	1.07 (1.03–1.11)	0.017	<0.001	1.00 (0.95–1.05)	0.026	0.967
Serum Potassium (mmol/L) 4^th^ (>4.67)	1.15 (1.11–1.19)	0.018	<0.001	0.96 (0.91–1.01)	0.027	0.096
**Calendar year**						
2006 (Referent Year)	1.00			1.00		
2007	1.03 (0.99–1.07)	0.021	0.199	1.07 (0.99–1.16)	0.042	0.109
2008	1.14 (1.09–1.19)	0.021	<0.001	1.37 (1.26–1.48)	0.041	<0.001
2009	1.16 (1.11–1.21)	0.022	<0.001	1.38 (1.27–1.49)	0.041	<0.001
2010	1.12 (1.08–1.17)	0.022	<0.001	1.35 (1.24–1.46)	0.042	<0.001
2011	1.17 (1.12–1.22)	0.022	<0.001	1.47 (1.35–1.59)	0.042	<0.001
2012	1.15 (1.10–1.20)	0.022	<0.001	1.47 (1.35–1.61)	0.046	<0.001
2013	1.18 (1.13–1.23)	0.022	<0.001	1.35 (1.23–1.47)	0.046	<0.001
2014	1.23 (1.18–1.28)	0.022	<0.001	1.44 (1.31–1.58)	0.047	<0.001

^1^Covariates for adjustment in the final multivariate model included: age group, sex, GFR category, county of residence, location of medical supervision, laboratory measures of illness (including haemoglobin, serum albumin, white blood cell count, serum potassium, serum calcium and serum phosphate concentrations) and calendar year.

Patients with the lowest levels of kidney function (eGFR categories 4 and 5) experienced the greatest likelihood of hyperuricaemia, OR, 10.92 (8.99–13.25) and 6.33 (4.58–8.74) in men and OR, 21.08 (16.99–26.16) and 8.66 (5.76–13.12) in women respectively as shown in Fig 5b. The adjusted OR of hyperuricaemia increased with each doubling of white cell count. Similarly, the adjusted OR of hyperuricaemia increased with rising serum phosphate and serum calcium concentrations. Increases in total serum protein concentrations were positively associated with hyperuricaemia, while increases in serum albumin concentrations exhibited a negative association. Patients with haemoglobin values in the highest quartile groups experienced the highest OR of hyperuricaemia compared to the referent group (2^nd^ quartile, haemoglobin 12.1–13.3 g/l).

### Geography and location of medical supervision with hyperuricaemia

The likelihood of hyperuricaemia varied significantly by county of residence. For example, male patients from Limerick county, OR, 1.33 (1.20–1.48) and Tipperary, OR, 1.34 (1.14–1.58) experienced significantly higher odds of hyperuricaemia compared to the referent county Donegal. The likelihood of hyperuricaemia was found to vary by location of medical supervision with a higher likelihood of hyperuricaemia found in all clinical settings compared to patients tested in the emergency room.

### Temporal trends in hyperuricaemia from 2006–2014

**Fig 6:**
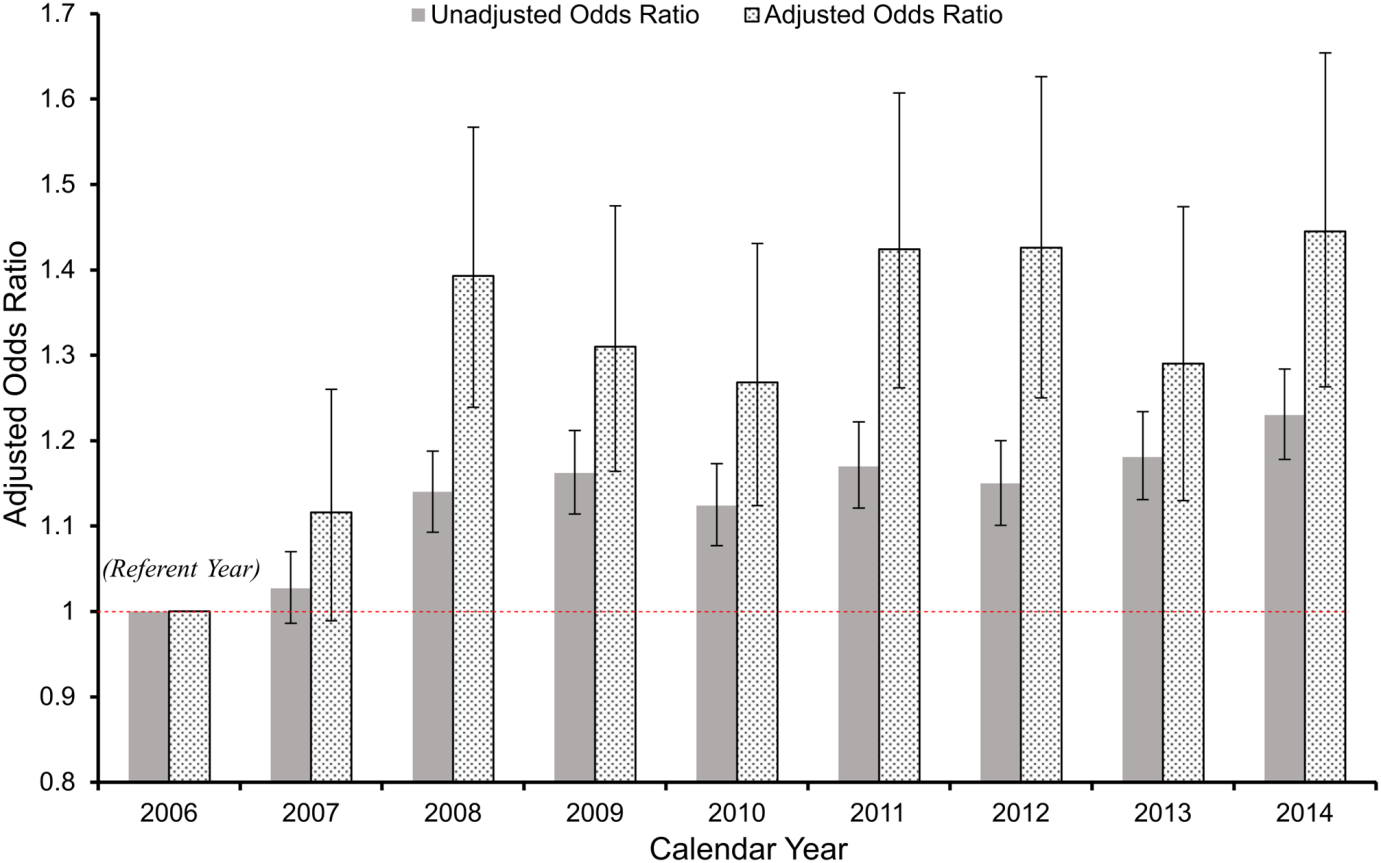
Univariable and multivariable odds-ratio (OR) for hyperuricaemia across calendar years in the Irish health system. In the multivariate model, covariates for adjustment include: age group, sex, eGFR CKD-stage in categories, county of residence, location of medical supervision, laboratory measures of illness (haemoglobin, serum albumin, white blood cell count, serum potassium, serum calcium and serum phosphate concentrations) and calendar year with referent 2006. P<0.001 for association of calendar year with 2006 as referent.

In univariate analysis, the OR of hyperuricaemia increased with successive calendar years as shown in Table 3 and Fig 6. With adjustment for confounding; patients who entered the health system in 2014 were significantly more likely to have hyperuricaemia compared to those in 2006 (referent), OR, 1.44 (1.31–1.58), P<0.001. This pattern of growth was consistent for men and women. Modelled as a continuous variable, the likelihood of hyperuricaemia increased by an average of 4% per year (95% CI 1.03–1.05), following adjustment. The final model had a c-statistic of 0.75 indicating good discrimination. In extended models (models 5 and 6), where we further adjusted for serum cholesterol, triglycerides and glycosylated haemoglobin, the pattern of association remained largely unchanged (Table 4).

**Table 4:** Odds ratio of hyperuricaemia between 2006 and 2014 in the Irish health system.

Model	Observations	2006	2007	2008	2009	2010	2011	2012	2013	2014	AUC
Unadjusted	249,821	1.00	1.03 (0.99–1.07)	1.14 (1.09–1.19)	1.16 (1.11–1.21)	1.12 (1.08–1.17)	1.17 (1.12–1.22)	1.15 (1.1–1.2)	1.18 (1.13–1.23)	1.23 (1.18–1.28)	0.52
Model 1*	249,821	1.00	1.02 (0.98–1.06)	1.11 (1.07–1.16)	1.12 (1.08–1.17)	1.07 (1.03–1.12)	1.11 (1.06–1.16)	1.08 (1.03–1.12)	1.09 (1.05–1.14)	1.12 (1.08–1.17)	0.60
Model 2¶	223,591	1.00	1.01 (0.97–1.06)	1.23 (1.18–1.28)	1.22 (1.17–1.28)	1.20 (1.14–1.25)	1.25 (1.19–1.3)	1.19 (1.14–1.25)	1.21 (1.15–1.26)	1.24 (1.18–1.29)	0.68
Model 3**	214,759	1.00	1.02 (0.98–1.07)	1.26 (1.21–1.32)	1.26 (1.2–1.31)	1.23 (1.17–1.28)	1.27 (1.21–1.33)	1.22 (1.16–1.27)	1.23 (1.17–1.28)	1.24 (1.19–1.3)	0.69
Model 4††	83,096	1.00	1.07 (0.98–1.16)	1.37 (1.26–1.48)	1.38 (1.27–1.49)	1.35 (1.24–1.46)	1.47 (1.35–1.59)	1.47 (1.34–1.61)	1.34 (1.23–1.47)	1.44 (1.31–1.58)	0.74
Model 5‡‡	28,129	1.00	1.16 (1.02–1.32)	1.58 (1.39–1.8)	1.54 (1.36–1.75)	1.61 (1.42–1.83)	1.62 (1.43–1.85)	1.55 (1.3–1.84)	1.54 (1.22–1.96)	1.50 (1.19–1.89)	0.75
Model 6†*	10,404	1.00	1.20 (0.95–1.52)	1.52 (1.22–1.9)	1.55 (1.24–1.93)	1.49 (1.19–1.87)	1.58 (1.26–1.97)	1.66 (1.27–2.16)	1.49 (1.15–1.93)	1.56 (1.21–2)	0.74

* Model 1: Age group and sex adjusted.

^¶^ Model 2: CKD stage, age group and sex adjusted.

** Model 3: County of Residence, Location of Medical Supervision, CKD stage, age group and sex adjusted.

^††^ Model 4: Additional adjustment for laboratory variables including (haemoglobin, white blood cell count, total protein, albumin, potassium, serum phosphate and calcium).

^‡‡^ Model 5: Model4 +additional adjustment for serum cholesterol and triglycerides.

^†^* Model 6: Model4 +HbA1c ≥6.5%.

### Sensitivity analysis

**Fig 7:**
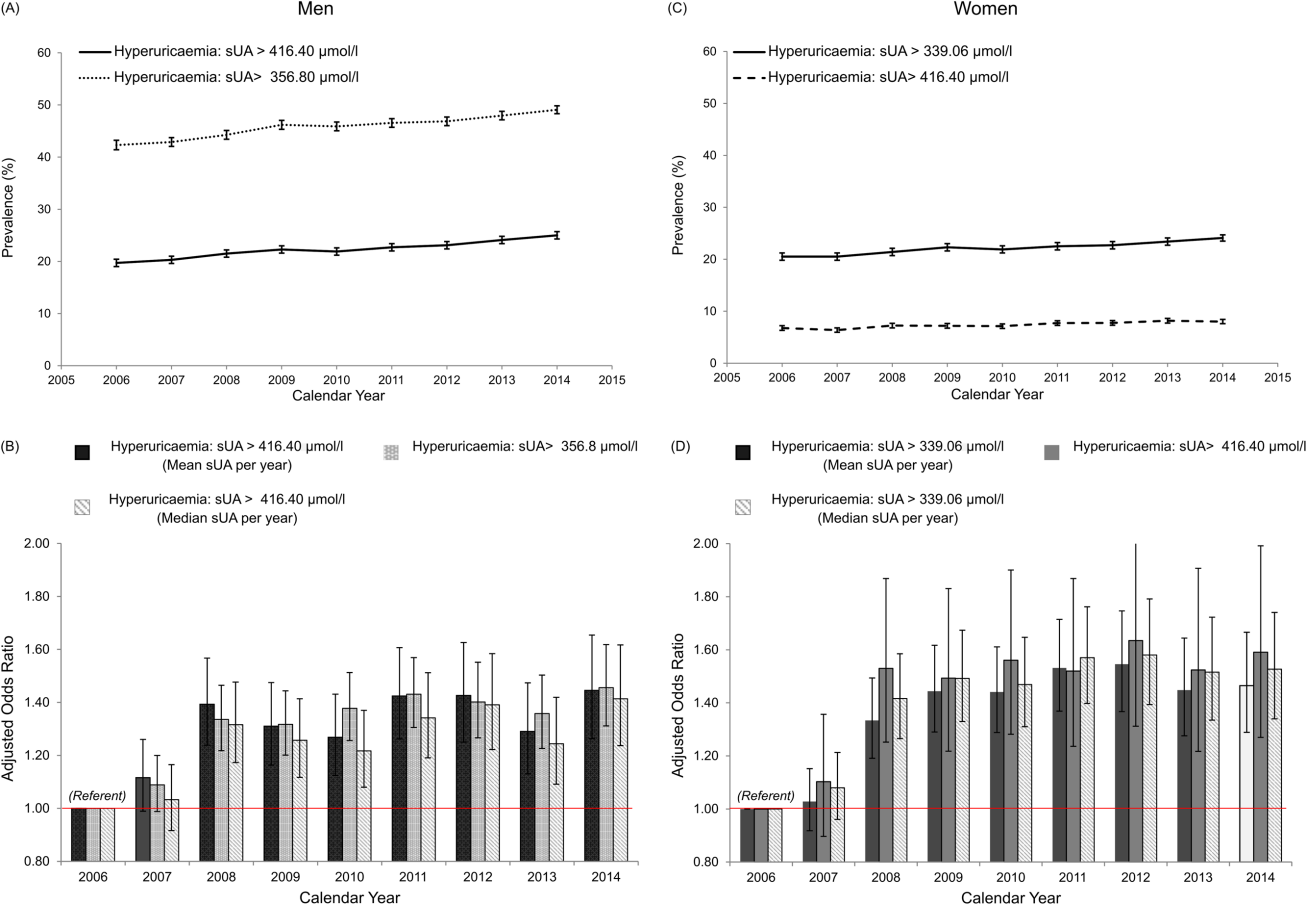
Sensitivity analyses: Multivariable odds-ratio (OR) for hyperuricaemia across calendar years in the Irish health system. (A) (B) Temporal trends in the prevalence of hyperuricaemia for sensitivity analyses 1 and 2 in men and women. (C) (D) In the multivariate model, covariates for adjustment include: age group, sex, an interaction term between age group and sex, eGFR CKD-stage in categories, county of residence, location of medical supervision, laboratory measures of illness (haemoglobin, serum albumin, white blood cell count, serum potassium, serum calcium and serum phosphate concentration) and calendar year with referent 2006. P<0.001 for association of calendar year with 2006 as referent.

The robustness of our findings was assessed in three sets of sensitivity analyses. First, using a lower sUA threshold of >356.8 mmol/l (6.0 mg/dl) based on EULAR targets. As expected, we demonstrated a higher prevalence of hyperuricaemia in men across successive years but the pattern of change that were similar to that from the main analysis Fig 7A. The association between calendar year and the likelihood of hyperuricaemia in men mirrored that of the primary analysis with an adjusted OR 1.45 (1.26–1.65), p<0.001 for hyperuricaemia in 2014 versus the referent year, 2006. Secondly, using a higher threshold value of UA >416.4 mmol/l (7.0 mg/dl), the prevalence of hyperuricaemia was correspondingly lower in women as shown in Fig 7B, but again this pattern of association mirrored that of the original analysis with an adjusted OR 1.40 (1.22–1.62), p<0.001 for 2014 versus referent year. Finally, on substituting the median for the mean sUA concentration, the same qualitative trends were observed for both men and women and are illustrated in Fig 7C and 7D.

## Discussion

In this large representative sample of patients within the Irish health System, we observed a substantial burden of hyperuricaemia and a pattern of increasing prevalence from 2006 to 2014. Overall, 20.1% of adult patients within the health system, men and women, were classified as having hyperuricaemia, a figure that increased to 24.5% in 2014. The net growth in prevalence of hyperuricaemia was 4.4% over the 9-year period and this pattern was consistent across health region, location of medical supervision, and across county of residence. Prevalence estimates were highest among elderly patients and those with severely impaired kidney function. Multivariable modelling found that the rise in annual prevalence was not accounted for by measured demographic characteristics, indicators of health status, or location of medical supervision. These findings collectively suggest that the burden of hyperuricaemia, a risk factor for gout and a risk marker for several metabolic conditions, is increasing in the Irish health system.

Uric acid, a metabolic waste product of purine metabolism, has been implicated in the genesis of several chronic medical conditions including gout, chronic kidney disease, hypertension, diabetes and death [2–9,11]. A fundamental requirement of any health system is surveillance of risk especially risk factors or potential risk factors that contribute to chronic disease, morbidity and mortality. Most published studies on hyperuricaemia prevalence to-date have based their analysis on national surveys, and therefore have not specifically addressed risk factor burden within the health system [10,12]. Moreover, analysis of country-specific trends has yielded conflicting results [10–12]. Data form the NHANES surveys in the US found that the prevalence of hyperuricaemia increased by 3.2% between 1988–1994 and 2007–2008. Similarly, data from the Health Search/CSD data base in Italy yielded a growth rate of 3.4% over a much shorter time span, 2005 to 2009. In contrast, results from consecutive Nutrition and Health Surveys in Taiwan (NAHSIT), covering the periods 1993–1996 to 2005–2008, have clearly shown a fall in prevalence, 3.3% in men and a remarkable 7.0% in women. Our results are concordant with findings from the US and Italian studies where hyperuricaemia increased by 4.4% over a 10 year-period and mean sUA concentrations increased from 314.6 to 325.6 μmol/L. We considered that the rise in prevalence of hyperuricaemia was driven by an increasing age profile, rising prevalence of CKD, or increasing burden of medical illness. However, in an analysis that adjusted for these changing demographic and clinical profiles, the pattern of growth persisted.

Our analysis uncovered striking differences in prevalence across representative demographic and clinical subgroups. It is noteworthy, that the highest prevalence was recorded in elderly patients (> 80 years), which was 3-fold higher than for patients in the 18–39 age group. Although young adults experienced the lowest prevalence of hyperuricaemia, they nevertheless experienced a significant growth of 4.9% over the 9-year period. An equally important finding was the observation that the prevalence of hyperuricaemia differed between men and women across age categories. Gender differences in the prevalence of hyperuricaemia were age-dependent. Among young adults, the prevalence was higher in men than in women, while in older patients, women experienced significantly higher prevalence than men. These findings would suggest that older women should be considered high-risk individuals for hyperuricaemia.

Patients with the poorest kidney function experienced the greatest burden of hyperuricaemia, highest for patients with eGFR values less than 30 ml/min/1.73m^2^. This is not surprising as impaired renal clearance of sUA is a major driver of hyperuricaemia [19–21]. Moreover, the phenotype of patients with CKD is likely to include the elderly, men, treatment with diuretics, gout and cardiovascular conditions [9,22]. When we accounted for age, gender and several clinical indicators of health status, we found that patients with the poorest kidney function (GFR values < 30mls/min/1.73m^2^) had the highest OR of hyperuricaemia. Despite this finding, our longitudinal analysis suggested that the prevalence of hyperuricaemia among patients with the poorest kidney function did not increase over time and in fact appeared to decrease, whereas the prevalence of hyperuricaemia increased significantly in all patients with eGFR values > 30 ml/min/1.73m^2^. This would suggest that either the risk factors that give rise to hyperuricaemia are better controlled in advanced CKD or that the use of ULT has increased for these high-risk CKD patients.

There are few limitations in our study that deserve mention. Our analysis of prevalence was based on estimates derived from patients within the health system and may not accurately reflect the true prevalence in the population. We also submit that sUA is not routinely measured in clinical practice which may affect the precision of the estimates. Furthermore while previous studies had adjusted for factors related to sUA levels (e.g. body mass index, hypertension, and diuretics) our study lacked data on these risk factors [10]. Despite these limitations, our study had several strengths. First, our dataset contained longitudinal data on all patients within a defined health system from 2006 to 2014 with serial measurements of sUA. Measurements of sUA were based on a single standardised assay across 2 regional laboratory systems reducing the risk of measurement error. The large sample size allowed us to carefully estimate prevalence with precision and map temporal trends in clearly defined demographic and clinical subgroups. Finally, although we lacked covariates on specific clinical and medication markers, our multivariable model was able to adjust for a wide set of demographic, geographic and laboratory measures of clinical health including kidney function.

## Conclusion

We found that the burden of hyperuricaemia is substantial in the Irish health system affecting almost a quarter of all adult men and women in 2014. We demonstrated a pattern of increasing prevalence from 2006 to 2014 that was present for most patient subgroups across multiple settings, with the exception of patients with advanced kidney impairment where a trend towards stabilisation was observed. Prevalence estimates were highest among elderly patients and those with severely impaired kidney function. The observed growth patterns were not accounted for by changing demographic and clinical profiles. These findings suggest that the burden of hyperuricaemia is increasing in the Irish health system. Given the emerging body of evidence linking hyperuricaemia to adverse clinical outcomes, better management of uric acid and its determinants should be a major goal.

## Supporting information

S1 Table Correlation structure criteria. (DOCX)
