# Supplementary material for: Temporal trends in hyperuricaemia in the Irish health system from 2006-2014: A cohort study
Source: PLoS One. 2018 May 31;13(5):e0198197. doi: 10.1371/journal.pone.0198197 (PMC5980488; doi:10.1371/journal.pone.0198197)
Supplement: S1 Table — (DOCX) [file pone.0198197.s001.docx]

.

Supplementary Table 1. Correlation Structure Criteria

|  | Independent Correlation Structure | Exchangeable Correlation Structure |
| --- | --- | --- |
| QIC | 75913.95 | 74.00 |
| CIC | 76168.13 | 69.16 |

The (correlation information criterion) CIC as proposed by Hin and Wang (2009) was used to assess which working correlation structure is appropriate. An independence model was fit to serve as a reference to the final multivariate model. The CIC for the exchangeable (CIC=69.16) is ranked better than the independence model (CIC=74.00) therefore an exchangeable correlation structure was chosen for the GEE as it better describes the correlation structure of the data.

..
